# Browning Effects of a Chronic Pterostilbene Supplementation in Mice Fed a High-Fat Diet

**DOI:** 10.3390/ijms20215377

**Published:** 2019-10-29

**Authors:** Martina La Spina, Eva Galletta, Michele Azzolini, Saioa Gomez Zorita, Sofia Parrasia, Marika Salvalaio, Andrea Salmaso, Lucia Biasutto

**Affiliations:** 1 Department of Biomedical Sciences, University of Padova, 35131 Padova, Italy; 2 Department of Biology, University of Padova, 35131 Padova, Italy; 3 Nutrition and Obesity Group, Department of Pharmacy and Food Science, University of the Basque Country (UPV/EHU) and Lucio Lascaray Research Institute, 01006 Vitoria, Spain; 4 CIBEROBN Physiopathology of Obesity and Nutrition, Institute of Health Carlos III, 01006 Vitoria, Spain; 5 Department of Pharmaceutical and Pharmacological Sciences, University of Padova, 35131 Padova, Italy; 6 Padova Unit, CNR Neuroscience Institute, 35131 Padova, Italy

**Keywords:** pterostilbene, 3T3-L1 adipocytes, C57BL/6 mice, browning, diet-induced obesity, high-fat diet

## Abstract

Obesity and related comorbidities are a major health concern. The drugs used to treat these conditions are largely inadequate or dangerous, and a well-researched approach based on nutraceuticals would be highly useful. Pterostilbene (Pt), i.e., 3,5-dimethylresveratrol, has been reported to be effective in animal models of obesity, acting on different metabolic pathways. We investigate here its ability to induce browning of white adipose tissue. Pt (5 µM) was first tested on 3T3-L1 mature adipocytes, and then it was administered (352 µmol/kg/day) to mice fed an obesogenic high-fat diet (HFD) for 30 weeks, starting at weaning. In the cultured adipocytes, the treatment elicited a significant increase of the levels of Uncoupling Protein 1 (UCP1) protein—a key component of thermogenic, energy-dissipating beige/brown adipocytes. In vivo administration antagonized weight increase, more so in males than in females. Analysis of inguinal White Adipose Tissue (WAT) revealed a trend towards browning, with significantly increased transcription of several marker genes (*Cidea*, *Ebf2*, *Pgc1α*, *PPARγ*, *Sirt1*, and *Tbx1*) and an increase in UCP1 protein levels, which, however, did not achieve significance. Given the lack of known side effects of Pt, this study strengthens the candidacy of this natural phenol as an anti-obesity nutraceutical.

## 1. Introduction

Obesity and related disorders are widely perceived by both the public and the scientific community as a major and growing threat to global human health; more than 2 billion people worldwide are currently overweight or obese [1], and obesity is associated with an increased hazard for a range of ailments, including type-2 diabetes, cardiovascular diseases and hypertension, multiple cancer types, chronic inflammatory diseases, dementia, and neurodegenerative diseases. All these complications lead to a shortened life expectancy and have a high economic impact on healthcare systems [2].

Lifestyle changes (such as diet modification, physical activity, and behavioral interventions) are the cornerstones of obesity treatment but maintaining a healthy lifestyle is often perceived as challenging. Alternatively, pharmacotherapy and bariatric surgery represent more “aggressive” interventions, and are applicable only if the problem is severe. Current pharmacotherapy for obesity relies on a few drugs [3] that are approved only for patients with BMI > 30. With the only exception of orlistat (which decreases fat absorption by inhibiting lipases), most of these drugs act through the central nervous system to reduce food intake (via reduction of appetite or enhancement of satiety). However, the use of these treatments may lead to severe adverse effects and a long-term prescription is usually not recommended.

A nutraceutical approach could be a useful supporting tool to treat overweight people, since the use of natural compounds offers clear advantages over drug treatments in terms of adverse side effects, suitability for chronic treatments, marketability, and safety [4]. Here we focus our attention on pterostilbene (Pt; 3,5-dimethylresveratrol), an analogue of resveratrol that differs from the latter for the presence of two methyl groups. Its increased lipophilicity accounts for its ability to reach higher tissue levels in comparison to resveratrol’s [5]. Moreover, there is no concern about its safety [6,7]. Pterostilbene has already been shown to have positive effects in combating obesity and related disorders [8]. In rat models of obesity, Pt reduces fat accumulation and ameliorates liver steatosis modulating lipolysis, lipogenesis, fatty acid oxidation [9,10,11,12,13], improves glycemic control by modulating glycolysis, gluconeogenesis, and glucose uptake [14,15], increases thermogenic markers in brown adipose tissue [16], and induces changes in the gut microbiota towards a healthier composition [17]. Furthermore, Pt exerts its beneficial effects combating adipose tissue inflammation [18].

An interesting therapeutic target for treating obesity and metabolic syndrome is represented by beige (or brite, i.e., brown-in-white) adipocytes. These cells are an inducible form of thermogenic adipocytes residing in subcutaneous white adipose tissue (WAT) and are also found in human adults [19,20,21,22]. Beige adipocytes can derive from white adipocyte trans differentiation and from the differentiation of adipogenic progenitor cells. They express a set of beige-specific genes, such as *Cited1* and *Tbx1* [19,21], but also have similarities with brown adipocytes, including abundant mitochondria, multilocular lipid droplets and expression of brown-specific genes such as *uncoupling protein 1* (*UCP1*) and *Cidea* [19]; similar to brown adipocytes, they are able to dissipate energy as heat. Induction of beige adipocytes (i.e., WAT browning) can be induced by environmental or physiological stimuli (such as cold exposure, physical activity, or thyroid hormones), but also pharmacologically (for example, with PPAR agonists [23]), and could prevent or combat obesity by increasing energy consumption through non-shivering thermogenesis [22,24].

Various phytochemicals have been reported to promote fat browning [25,26,27,28,29]. The aim of this study was to investigate the ability of Pt to do it in WAT. Its effects were first assayed on cultured 3T3-L1 adipocytes, and then in a mouse model of diet-induced obesity. To take into account possible gender-specific differences, both females and males were included in our experimental groups; a 30 week-long treatment was performed, to highlight long-term effects of a chronic Pt supplementation. We found that Pt diminished body weight gain induced by a high-fat diet regimen; also, glucose homeostasis was partially preserved, at least up to week 18. Interestingly, Pt was able to induce WAT browning, leading to an increase in the transcription of beige- and brown-related genes and of UCP1 protein levels, which, however, did not achieve significance.

## 2. Results

### 2.1. Choice of Pt Dosage

**Figure 1:**
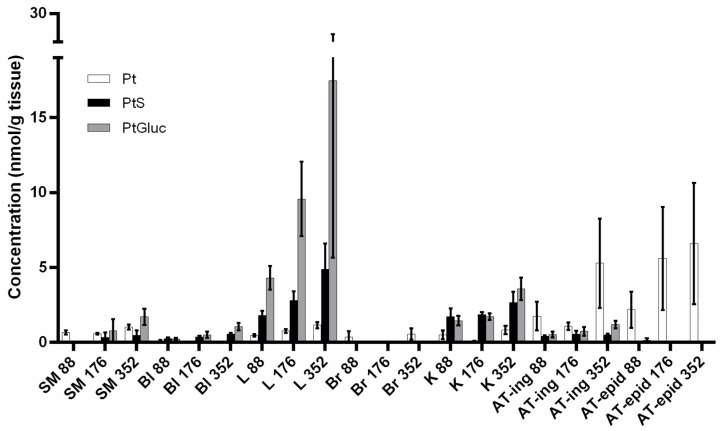
Tissue distribution of pterostilbene (Pt) and its main metabolites Pt-sulfate (PtS) and Pt-glucuronide (PtGluc) after chronic oral administration of Pt for 2 weeks at three different dosages: 88, 176, and 352 µmol/kg/day. SM = skeletal muscle; Bl = blood; L = liver; Br = brain; K = kidney; AT-ing = inguinal adipose tissue; AT-epid = epididymal adipose tissue. *n* = 4. Mean values ± SEM are shown; these data are also tabulated in Table S1.

To define the most suitable dose of pterostilbene to be used during in vivo experiments, we performed preliminary tissue distribution experiments. Mice were fed with a high fat diet (HFD) supplemented with three different dosages of pterostilbene for 2 weeks (see Materials and Methods for details). The higher dosage (352 µmol/kg/day, corresponding to 90 mg/kg/day) was selected to be the most suitable one, since it allowed the achievement of µM levels of the compound in all major organs and also in both epididymal and inguinal adipose tissues (Figure 1 and Table S1).

### 2.2. Pt Effect on 3T3-L1 Mature Adipocytes

The ability of Pt to increase the expression of (at least some) browning genes was tested in vitro on cultured 3T3-L1 adipocytes; cells were grown and differentiated following standard experimental protocols [9]. On day 12 they can be considered fully differentiated and they showed typical accumulation of large lipid droplets in the cytoplasm. Mature 3T3-L1 adipocytes were incubated for 24 h with 5 µM pterostilbene, which is the mean concentration we observed in both epididymal and inguinal adipose tissues after chronic administration of 352 µmol/kg/day Pt to mice.

**Figure 2:**
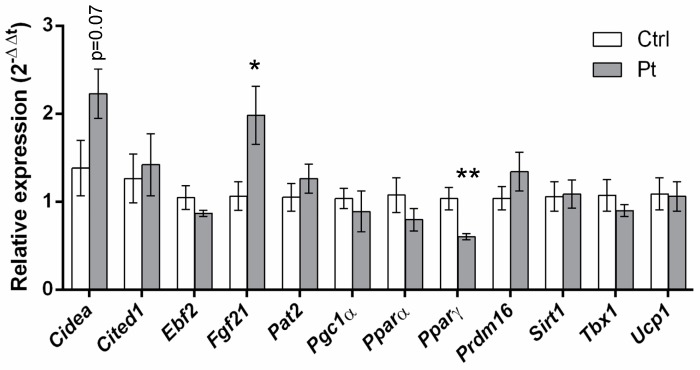
Gene expression levels in 3T3-L1 adipocytes. Mean values ± SEM are shown. *n* = 6 for each condition. *: *p* ≤ 0.05; **: *p* ≤ 0.01.

**Figure 3:**
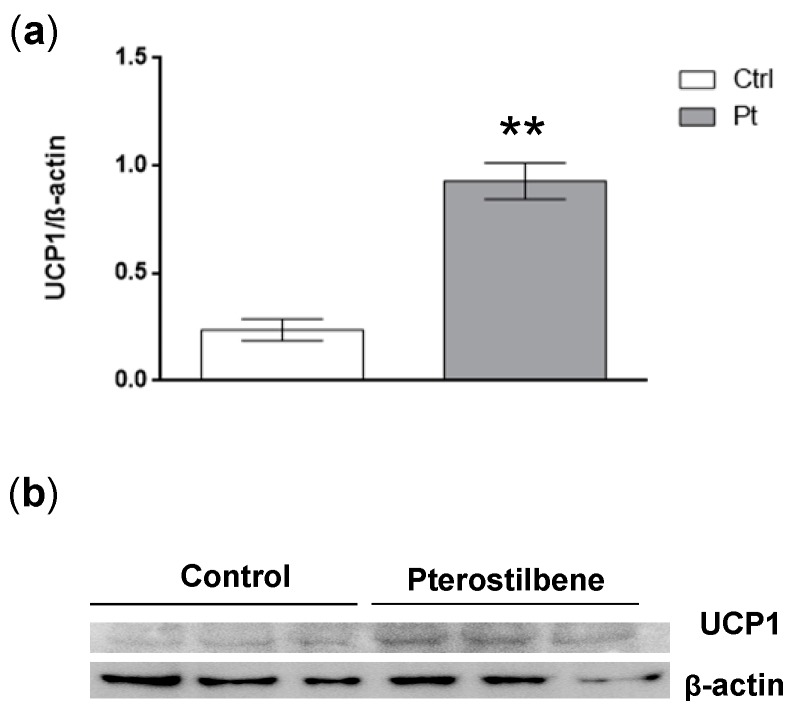
Uncoupling protein 1 (UCP1) in mature 3T3-L1 adipocytes. (**a**) Western Blot quantification; protein levels were normalized to β-actin. Mean values ± SEM are shown. *n* = 3 for each condition. (**b**) Representative image of Western Blot protein bands. **: *p* ≤ 0.01.

RT-qPCR of 3T3-L1 adipocytes showed an increase in the transcription of *Cidea* and *Fgf21* and a decrease in *PPARγ* after Pt treatment. *Ucp1* transcript levels were not affected (Figure 2), but UCP1 protein levels were increased (Figure 3).

### 2.3. Pt Effect on Body Weight Increase

**Figure 4:**
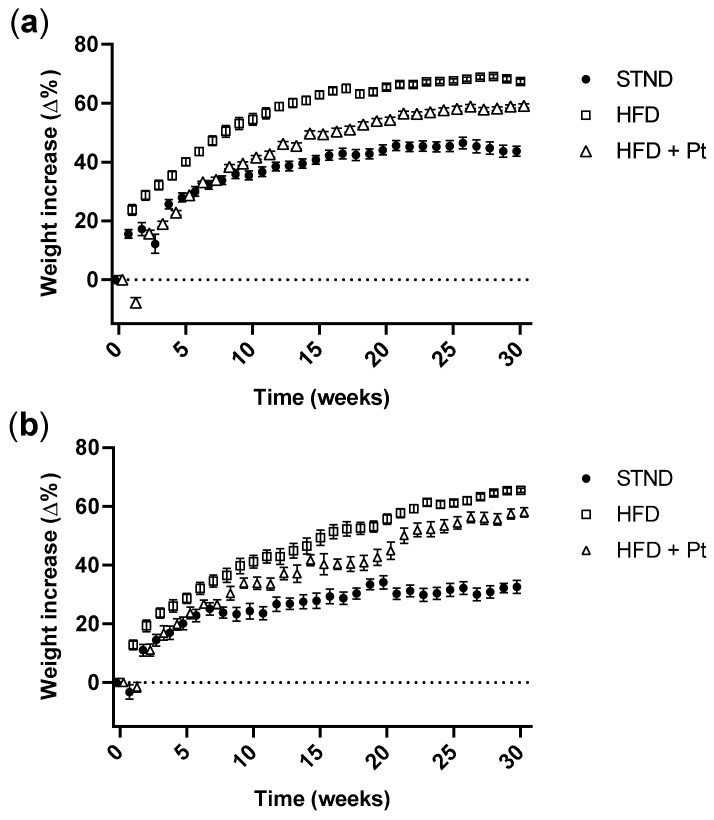
Body weight increase of (**a**) male and (**b**) female mice fed a standard diet (STND), a high fat diet (HFD), or a high fat diet supplemented with Pt (HFD + Pt). Data are expressed as percentage of body weight increase compared to body weight at each time point. *n* ≥ 7 for each condition, mean values ± SEM.

Weight gain of the mice receiving the HFD from weaning was higher compared to that of mice fed a standard diet (*p* ≤ 0.05 at all time points for both females and males). Notably, the administration of Pt slowed down weight increase; the effect was somewhat different between males and females. Both in males and females, for the first 8 weeks of treatment the body weight increase of the HFD + Pt group was not significantly different from that of the standard diet (STND) group. From week 9 (and until the end of the experiment), the weight increase of HFD + Pt groups became significantly higher than that of STND mice (*p* ≤ 0.01 and *p* ≤ 0.05 at all time points for males and females, respectively), but for males the curve remained also significantly lower than the HFD group (*p* ≤ 0.002 at all time points) (Figure 4a). On the other hand, the body weight increase of HFD + Pt females became more similar to that of the HFD group (differences were not significant at weeks 9, 10, 12–15, 21, and 22) (Figure 4b).

### 2.4. Glucose and Insulin Tolerance Tests (GTT, ITT)

**Figure 5:**
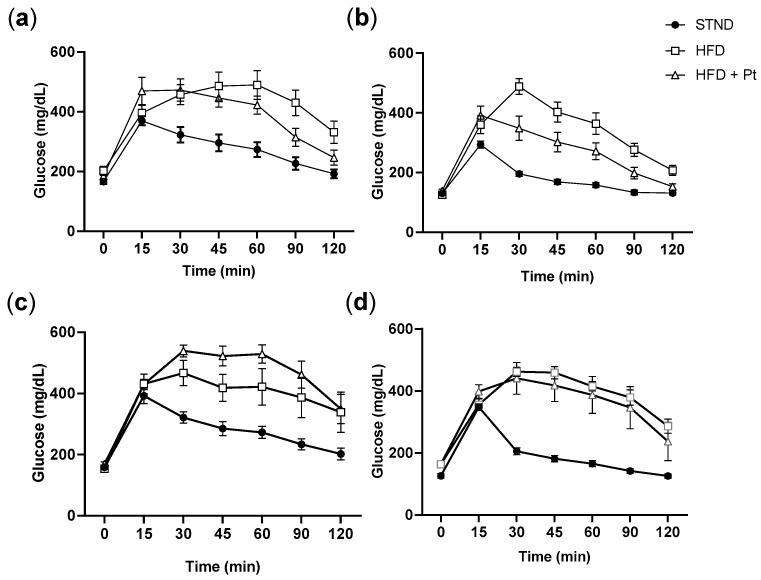
Glucose tolerance in (**a**,**c**) male and (**b**,**d**) female mice. Experiments were performed after (**a**,**b**) 18 weeks or (**c**,**d**) 28 weeks from the beginning of the high-fat diet regimen. *n* ≥ 7 for each condition; mean values ± SEM.

The glucose tolerance test was performed in fasted animals after 18 and 28 weeks of treatment. Altered glucose homeostasis was observed in both males and females of the HFD group at both times (Figure 5). Pt treatment slightly improved glucose homeostasis in obese mice, but only after 18 weeks of treatment. For both males and females, glucose levels at 120 min were not significantly different from those measured in the corresponding STND groups (Figure 5a,b). At 28 weeks this effect was no longer observed, and no significant differences were detected between HFD and HFD + Pt groups (Figure 5c,d).

The insulin tolerance test was also performed at 18 and 28 weeks. No significant effects were associated with Pt supplementation in any case (Figure S1).

### 2.5. Pt Effect on Inguinal WAT

**Figure 6:**
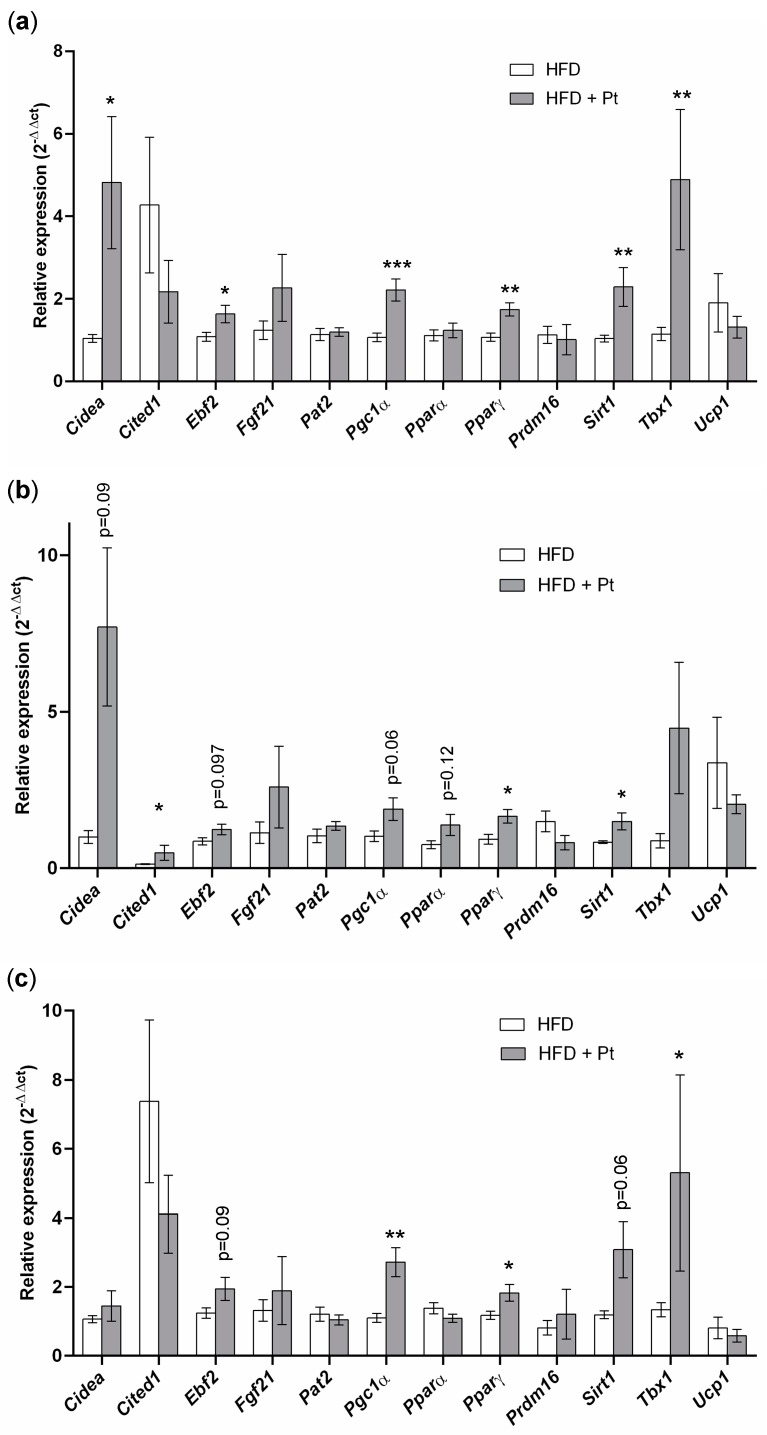
Gene expression analysis of inguinal white adipose tissue from HFD and HFD + Pt mice. Data from (**a**) males and female averaged together; (**b**) males; (**c**) females. Mean values ± SEM are shown. *n* ≥ 5 for each condition. *: *p* ≤ 0.05; ** *p* ≤ 0.01; ***: *p* ≤ 0.001.

**Figure 7:**
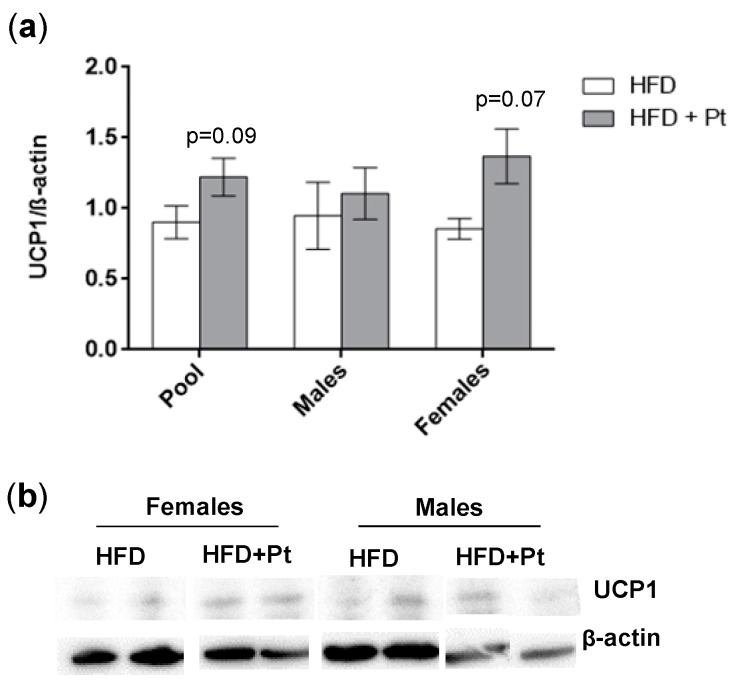
UCP1 protein levels in inguinal white adipose tissue of HFD and HFD + Pt mice. (**a**) Western Blot quantification. Protein levels were normalized to β-actin. Mean values ± SEM are shown. Data from males and females were pooled together (*n* ≥ 8 for each condition) or considered separately (*n* ≥ 4 for each condition). (**b**) Representative image of Western Blot protein bands.

**Figure 8:**
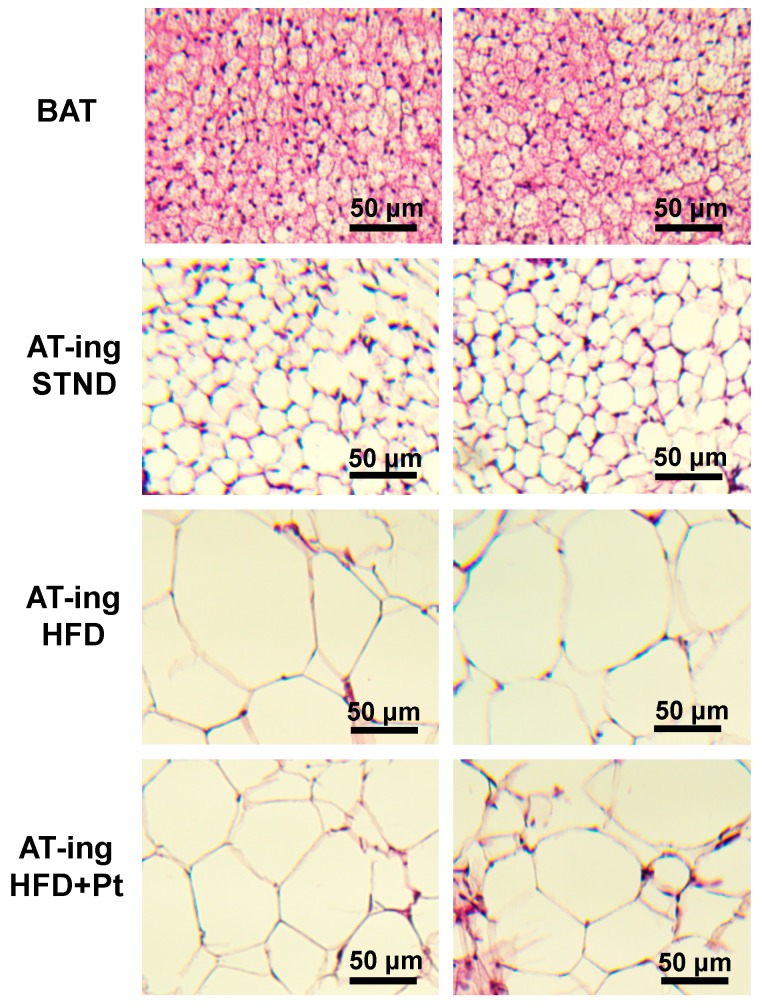
Representative sections (hematoxylin-eosin staining) of interscapular brown adipose tissue (BAT) and inguinal white adipose tissue (AT-ing) from STND, HFD or HFD + Pt animals. Pictures were captured at 10X magnification.

We analyzed the effect of Pt treatment on inguinal WAT since this is known from literature to be the WAT depot most prone to undergo browning in mice [30]. RT-qPCR analysis showed a general increase of browning markers in the HFD + Pt group, compared to the HFD one: a significant increase (*p* ≤ 0.05) in transcript levels was observed for *Cidea*, *Ebf2*, *Pgc1a*, *PPARγ*, *Sirt1*, and *Tbx1* (Figure 6a). This trend towards browning was also observed considering data from males and females separately (Figure 6b,c). In accordance with our in vitro experiments, *Ucp1* mRNA levels did not change with Pt supplementation, while its protein expression seems to be upregulated (*p* = 0.09; Figure 7a). Interestingly, if data from males and females are considered separately, Pt seems to affect females (*p* = 0.07; Figure 7) more than males (*p* = 0.62; Figure 7). Histological analysis on a representative set of samples showed a slight decrease in adipocyte size in the HFD + Pt group compared to the HFD one (Figure 8).

## 3. Discussion

The recent finding that browning can also take place in human adults [19,20,21,22] pointed to browning of white adipose tissue as a promising strategy to prevent and/or treat obesity and related comorbidities, such as type 2 diabetes [29].

A nutraceutical approach to the field could offer various advantages compared to pharmacotherapy, for example in terms of applicability (i.e., also used for obesity prevention and for “mild” cases), absent/limited side effects and toxicity, and suitability for chronic/long-term administrations. Pterostilbene, the resveratrol analog studied in this work, has been demonstrated to exert multiple beneficial effects on obesity [9,11,14,15,16]. Until now, however, there was no evidence on whether Pt could exert beneficial effects by inducing browning in white adipose tissue. In this work we thus tested Pt for its ability to induce WAT browning in a mouse model of diet-induced obesity.

Pt effects were evaluated looking at the transcription levels of a panel of genes that are beige-specific (*Cited1*, *Tbx1*) [19], brown-related (*Cidea*, *Prdm16*, *Ucp1*) [19], or are involved in the regulation of the browning process (*Sirt1* [31,32,33], *PPARα* [34], *PPARγ* [34,35,36], *Pgc1α* [24], *Ebf2* [37], *Fgf21* [38]) (rev: [28]).

We first of all assessed Pt effects on cultured mature 3T3-L1 adipocytes, and observed that Pt treatment was only able to upregulate the expression of *Fgf21*, a key factor regulating browning [38], and the brown-specific gene *Cidea*. On the other hand, Pt significantly decreased expression of *PPARγ*; this transcription factor may play different roles, depending on its association with different cofactors and the formation of different transcriptional complexes. Dynamic interactions, as well as epigenetic modifications, further modulate its transcriptional activity [39], which can eventually promote differentiation to brown adipocytes but also induce adipogenesis [40]. It is already reported in the literature that a pharmacological stimulation (i.e., treatment with a beta-adrenergic receptor agonist) of 3T3-L1 cells to induce browning did not actually change transcription levels of beige-selective genes, and induced a decrease of *PPARγ* expression [41].

Protein levels of UCP1 were significantly increased by Pt in 3T3-L1 cells, although no differences were observed at the transcriptional level. This discrepancy between gene and protein expression was also observed by other groups in other models [42], and could be related, for example, to protein turnover regulation [43,44].

The browning effect of Pt was then assessed by analyzing the inguinal white adipose tissue of mice. Compared with mice fed the high fat diet only, a 30 week treatment with Pt induced an increase in the expression of various genes regulating the browning process, such as *Ebf2*, *Sirt1*, *Pgc1α*, and *PPARγ*. *Ebf2* has been shown to be necessary and sufficient to stimulate beige adipocytes development in the WAT of mice [37]; *Ebf2* was also found to be selectively expressed in brown adipocytes, where it binds to brown adipose-specific PPARγ target genes [45]. Sirt1 has been shown to deacetylate PPARγ on different lysine residues, thus facilitating its docking with PRDM16 and PGC1α, and finally stimulating the transcription of brown-adipocyte specific genes [28,32]. Moreover, Sirt1 activates PGC1α, promoting mitochondriogenesis and oxidative metabolism [28,46]. PPARγ has been reported to be the master regulator for the differentiation of white and brown adipocytes, and PPARγ agonists were reported to induce the expression of Ucp1 not only in animals but also in humans [23]. Evidence of PPARγ activation in HFD + Pt animals was suggested by the increased transcription of *Cidea*, which is a PPARγ target gene (http://www.ppargene.org/) and is related to browning.

In conjunction with these results, we also observed an upregulation of the beige-specific gene *Tbx1*.

As observed in 3T3-L1 adipocytes, in vivo Pt treatment did not affect *Ucp1* transcript levels, whereas it increased UCP1 protein levels.

One relevant aspect in this work was the inclusion in the experimental groups of both males and females. Gender-specific effects are an important aspect, and have to be taken into account considering, for example, that brown adipocytes express sex steroid hormone receptors such as androgen, progesterone, and estrogen receptors [47]. Looking at the effects of Pt considering males and females separately, we observed a trend in the reduction of body weight increase in both the HDF + Pt groups, more so in males than in females; Pt-induced changes in browning-related gene expression also showed a similar trend in both males and females. On the other hand, protein levels of UCP1 seem to be upregulated by Pt treatment only in females (*p* = 0.07). Female mice have been reported to be more responsive than males to browning [48], and estradiol (E2) has been reported to influence WAT browning through different mechanisms [48,49,50,51,52].

The results we obtained confirm that pterostilbene may be a valid co-adjuvant in the fight against obesity and metabolic syndrome. The design of the study involved co-administration with the high-fat diet from weaning. One may predict the molecular effects to be largely the same if a protocol involving administration to already obese individuals were used. This however needs to be experimentally verified, especially since these circumstances would be the most relevant for a possible intervention in humans.

## 4. Materials and Methods

### 4.1. 3T3-L1 Cell Culture and Differentiation

3T3-L1 preadipocytes were cultured in DMEM containing 10% fetal bovine serum (FBS) and 1% penicillin/streptomycin (10,000 U/mL). Two days after confluence (day 0 of differentiation), the cells were stimulated to differentiate by the addition into the culture medium of 1% biotin, 1% panthothenic acid, 10 μg/mL insulin, 0.5 mM isobutylmethylxanthine (IBMX), and 1 μM dexamethasone. After 2 days, the differentiation medium was replaced by a medium containing FBS, penicillin, streptomycin, 1% biotin, 1% pantothenic acid, and 10 μg/mL insulin, and from day 4 onward by the same medium containing 0.2 μg/mL insulin (instead of 10 μg/mL). This medium was changed every 2 days until cells were harvested. Cells were maintained at 37 °C in a humidified 5% CO_2_ atmosphere. For the 3T3-L1 adipocyte treatment, cells grown in 6-well plates were incubated with pterostilbene 5 μM (0.1% final DMSO) for 24 h on day 12 after differentiation. By that day, >90% of cells were mature adipocytes with visible lipid droplets.

### 4.2. Animals

C57BL/6 mice were housed in the facility of the Department of Pharmaceutical and Pharmacological Sciences (University of Padova, Padova, Italy); food and water were provided ad libitum. All procedures were approved by the University of Padova Ethical Committee for Animal Welfare (OPBA) and by the Italian Ministry of Health (Permit Number 211/2015-PR, approved on 7 April 2015), and conducted with the supervision of the Central Veterinary Service of the University of Padova, in compliance with Italian Law DL 26/2014, embodying UE Directive 2010/63/EU (approved on 14 March 2014).

### 4.3. Tissue Distribution Studies

Preliminary tissue distribution studies were performed to assess the most suitable dose of pterostilbene to be used. Mice were fed for 2 weeks with a high-fat diet (60% calories from fat; OpenSource Diets, cat. n. D12492) supplemented with pterostilbene (Wonda Science, Changzhou, China; cat. n. 25992) (88, 176, or 352 µmol/kg body weight/day, respectively). At the end of the treatment, animals were anesthetized with isoflurane and sacrificed (*n* = 4 for each condition). Blood was collected in heparinized tubes, kept in ice and treated within 10 min. Skeletal muscle (gastrocnemius), liver, brain, kidneys, visceral adipose tissue, and subcutaneous adipose tissue were explanted, weighed, and immediately frozen in liquid nitrogen. Pterostilbene, pterostilbene-sulfate, and pterostilbene-glucuronide were extracted from blood and major organs (skeletal muscle, liver, brain, and kidneys) as described in [5]. Recovery yields of pterostilbene-glucuronide from blood and tissues were assumed to be the same as for pterostilbene-sulfate [5]. Adequate recovery yields from adipose tissue were obtained modifying this extraction protocol, as described in [53]. Analytes in the extracts were finally quantified through HPLC/UV analysis (see below, Section 4.4).

### 4.4. HPLC/UV Analysis

Samples (2 µL) were analyzed by HPLC/UV (1290 Infinity LC System, Agilent Technologies, Cernusco sul Naviglio (Milano), Italy) using a reverse phase column (Zorbax Extend-C18, 1.8 µm, 50 × 3.0 mm i.d.; Agilent Technologies, cat. n. 727975-302) and a UV diode array detector (190–500 nm). Solvents A and B were water containing 0.1% trifluoroacetic acid (TFA) and acetonitrile, respectively. The gradient for B was as follows: 10% for 0.5 min, then from 10% to 100% in 3.5 min, 100% for 1 min; the flow rate was 0.6 mL/min and the column compartment was maintained at 35 °C. Eluate was preferentially monitored at 286, 300, and 320 nm (corresponding to absorbance maxima of the internal standard, derivatives/metabolites, and pterostilbene, respectively).

### 4.5. Animal Treatments

After weaning (about 5 weeks from birth), C57BL/6 mice were randomly divided into three 16 member groups, each containing an equal number of males and females: STND, receiving a standard diet; HFD, receiving a high-fat diet (60% calories from fat; OpenSource Diets, New Brunswick, NJ, USA; cat. n. D12492); HFD + Pt, receiving a high-fat diet supplemented with pterostilbene (352 µmol/kg body weight/day). Mice were maintained under these three different dietetic regimens for 30 weeks.

Body weight was measured weekly; glucose and insulin tolerance were monitored twice (after 18 and 28 weeks; see Section 4.6 and Section 4.7) in order to evaluate the establishment of the diet-induced obesity phenotype and the efficacy of the Pt treatment.

After 30 weeks, animals were fasted for 4 h and then sacrificed. Inguinal adipose tissue was collected, immediately frozen in liquid nitrogen, and then stored at −80 °C.

### 4.6. Glucose and Insulin Tolerance Tests (GTT and ITT)

GTT and ITT were performed after 18 and 28 weeks of treatment. Animals were fasted for 6 h prior to the assay. Blood was collected from the tail vein, and blood glucose levels were measured with an Accu-Chek glucometer (Roche Diagnostics, Monza, Italy) at time 0 and 15, 30, 45, 60, 90, and 120 min after an intraperitoneal injection of D-glucose (1 g/kg) or of bovine insulin (0.75 U/kg; from Sigma-Aldrich, Milano, Italy; cat. n. I0516).

### 4.7. RNA and Protein Extraction

The frozen inguinal adipose tissue from control and treated mice was pulverized in liquid nitrogen using a mortar and a pestle, and the resulting frozen powder was divided evenly into two test tubes, for RNA and protein extraction respectively.

Total RNA was extracted using TRIzol reagent (Thermo Fisher, Waltham, MA, USA) according to the manufacturer’s instructions, adding a centrifugation step (5 min at 12,000× *g* at 4 °C) after TRIzol lysing to eliminate most of the fat content of the tissue.

Protein extraction was performed by resuspending 3T3-L1 cells (washed twice with PBS) or the frozen tissue powder in RIPA lysis buffer containing protease inhibitors, incubating for 30 min on ice, and then physically disaggregating the tissue with a potter. The lysate was then centrifuged at 20,000× *g* for 15 min at 4 °C to remove fat and transferred to a new tube.

### 4.8. Quantitative Real Time PCR

From the adipose tissue, 400 ng of total RNA was retrotranscribed with the Superscript VILO reverse transcriptase kit (Thermo Fisher) according to the manufacturer’s protocol, and the expression levels of the genes of interest were analyzed by quantitative real time PCR, using the iQ SYBR Green Supermix (Bio-Rad, Segrate, Italy) kit on a Bio-Rad CFX96 Real-Time System and C1000 Touch Thermal Cycler instrument. All samples were run in triplicate, and the housekeeping gene *GAPDH* was used as an internal control to normalize the expression levels, applying the 2^−ΔΔ*C*t^ method [54]. The primers used were designed using the Primer3 software, and all the sequences can be found in Table S2.

### 4.9. Western Blot

Proteins were separated by SDS-PAGE (Pre-cast NuPAGE Bis-Tris Gels, Invitrogen-Life Technologies, Monza, Italy). After electrophoretic separation, proteins were transferred to PVDF membranes (immobilion-FL). The membranes were saturated with 5% BSA (Sigma-Aldrich) in TBS-T buffer (TBS supplemented with 0.1% Tween-20; Sigma Aldrich) for 1 h and then incubated overnight at 4 °C with the primary antibody. Primary antibodies used (all from Cell Signaling, Danvers, MA, USA) were anti-β-actin (#8457S) and anti-UCP1 (#14670). Secondary goat anti-rabbit antibody (Cell Signaling, #7074) was horseradish peroxidase-conjugated and was used with chemiluminescence detection (Pierce) using digital imaging by a UVITEC Eppendorf apparatus.

### 4.10. Histological Analysis

Immediately after excision, adipose tissue samples were fixed in 4% paraformaldehyde (overnight, 4 °C). They were then sequentially transferred in a 30% ethanol solution (EtOH; 30 min), followed by 50% EtOH (30 min), then 70% EtOH; samples were then stored in a 70% EtOH solution at 4 °C, until paraffin embedding.

Paraffin embedding: samples were sequentially transferred in 70% EtOH (30 min), 95% EtOH (1 h), 100% EtOH (1 h; twice), xylene (1.5 h; twice), then paraffin (1.5 h, 60 °C; four times), and they were finally embedded in paraffin.

Tissue was then sliced into 8 µm thick sections with a microtome, mounted on coverslips, and stained with hematoxylin/eosin.

Hematoxylin/eosin staining: slides were sequentially immersed in xylene (15 min), 100% EtOH (10 min), 95% EtOH (10 min), 70% EtOH (10 min), 50% EtOH (10 min), distilled water (5 min), ematoxylin (10 min), tap water (10 min), distilled water (2 min), 1% eosin (5 min), tap water (5 min), 50% EtOH (2 s), 70% EtOH (2 s), 95% EtOH (2 s), 100% EtOH (2 s), xylene (10 min), and were finally mounted and examined with a Leitz Dialux 22 microscope, equipped with an OPTIKA microscopes C-P8 camera, using a 10X objective.

### 4.11. Statistical Analysis

Statistical analysis was performed using GraphPad Prism Software, version 6. Significance in comparisons was assessed using Student’s t-test (changes in gene expression or protein levels) or two-way ANOVA for repeated measures (body weight increase, GTT curves). Tukey’s test was used for multiple comparisons.

## Supplementary Materials

Supplementary materials can be found at https://www.mdpi.com/1422-0067/20/21/5377/s1.
