# Supplementary material for: Browning Effects of a Chronic Pterostilbene Supplementation in Mice Fed a High-Fat Diet
Source: Int J Mol Sci. 2019 Oct 29;20(21):5377. doi: 10.3390/ijms20215377 (PMC6862528; doi:10.3390/ijms20215377)
Supplement: Supplementary file 1 [file ijms-20-05377-s001.pdf]

Supplementary Material

Browning Effects of a Chronic Pterostilbene Supplementation in Mice Fed a High-Fat Diet

Martina La Spina [1,†,‡], Eva Galletta [1,2,†], Michele Azzolini [1,§], Saioa Gomez Zorita [3,4], Sofia Parrasia [1], Marika Salvalaio [5], Andrea Salmaso [1,6] and Lucia Biasutto [1,6,*]

1 Department of Biomedical Sciences, University of Padova, 35131 Padova, Italy
2 Department of Biology, University of Padova, 35131 Padova, Italy
3 Nutrition and Obesity Group, Department of Pharmacy and Food Science, University of the Basque Country (UPV/EHU) and Lucio Lascaray Research Institute, 01006 Vitoria, Spain
4 CIBEROBN Physiopathology of Obesity and Nutrition, Institute of Health Carlos III, 01006 Vitoria, Spain
5 Department of Pharmaceutical and Pharmacological Sciences, University of Padova, 35131 Padova, Italy
6 Padova Unit, CNR Neuroscience Institute, 35131 Padova, Italy
* Correspondence: lucia.biasutto@cnr.it
† These authors contributed equally to this work
‡ Current address: Cell Biology and Physiology Center, National Heart, Lung and Blood Institute, National Institutes of Health, Bethesda, MD 20892, USA.
§ Current address: Department of Physiology and Pharmacology, Karolinska Institutet, 17177 Stockholm, Sweden

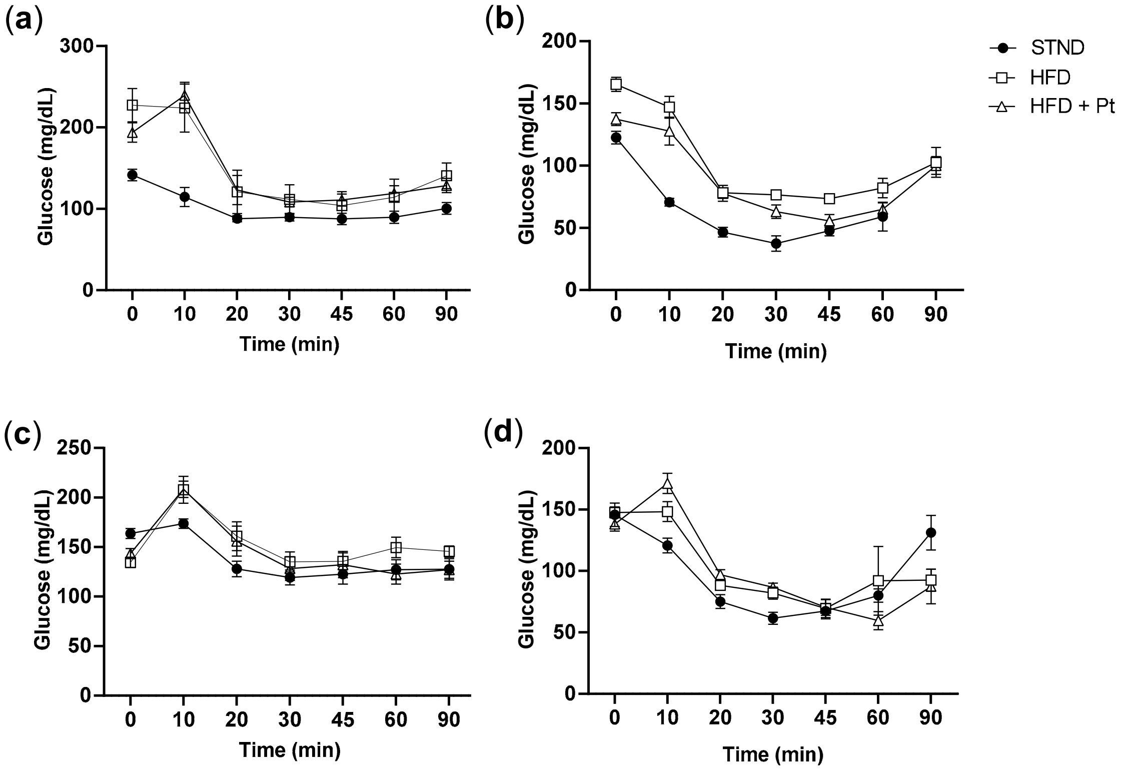

Figure S1. Insulin tolerance tests in (**a**), (**c**) male and (**b**), (**d**) female mice. Measurements were performed after (**a**), (**b**) 18 weeks or (**c**), (**d**) 28 weeks from the beginning of the high-fat diet regimen. N ≥ 7 for each condition; mean values ± SEM.

Table S1. Tissue levels of Pt, Pt-sulfate (PtS) and Pt-glucuronide (PtGluc) after chronic administration of Pt at three different dosages. N = 4, mean values ± SEM.

**Tissue**	**Dosage** (µmol/kg/day)	**Pt** (nmol/g)	**PtS** (nmol/g)	**PtGluc** (nmol/g)
**SM**	88	0.68 ± 0.14	0	0
	176	0.59 ± 0.05	0.34 ± 0.34	0.77 ± 0.77
	352	1.03 ± 0.17	0.47 ± 0.36	1.71 ± 0.54
**Bl**	88	0.11 ± 0.06	0.25 ± 0.09	0.25 ± 0.08
	176	0	0.36 ± 0.07	0.51 ± 0.19
	352	0	0.57 ± 0.09	1.06 ± 0.25
**L**	88	0.48 ± 0.08	1.81 ± 0.32	4.31 ± 0.79
	176	0.77 ± 0.12	2.82 ± 0.59	9.58 ± 2.48
	352	1.15 ± 0.22	4.87 ± 1.71	17.47 ± 11.81
**Br**	88	0.37 ± 0.37	0	0
	176	0	0	0
	352	0.56 ± 0.38	0	0
**K**	88	0.51 ± 0.30	1.73 ± 0.55	1.46 ± 0.33
	176	0.05 ± 0.05	1.87 ± 0.17	1.72 ± 0.21
	352	0.83 ± 0.28	2.67 ± 0.71	3.58 ± 0.75
**At-ing**	88	1.76 ± 0.96	0.43 ± 0.05	0.53 ± 0.20
	176	1.08 ± 0.25	0.56 ± 0.21	0.75 ± 0.29
	352	5.29 ± 2.98	0.50 ± 0.08	1.21 ± 0.25
**At-epid**	88	2.18 ± 1.21	0	0.14 ± 0.14
	176	5.62 ± 3.44	0	0
	352	6.61 ± 4.04	0	0

[1] SM = skeletal muscle; Bl = blood; L = liver; Br = brain; K = kidney; AT-ing = inguinal adipose tissue; AT-epid = epididymal adipose tissue.

Table S2. List and sequences of the primers used for rtPCR analysis.

Gene	Primer
Cidea	F: GCCGTGTTAAGGAATCTGCTG<br>R: TGCTCTTCTGTATCGCCCAGT
Cited1	F: ATTTATCGGACTTCTGCCCAG<br>R: TTGCGATCCTTCACTCCAAG
Ebf2	F: GGGATTCAAGATACGCTAGGAAG<br>R: GGAGGTTGCTTTTCAAAATGGG
Fgf21	F: CAAATCCTGGGTGTCAAAGC<br>R: CATGGGCTTCAGACTGGTAC
Gapdh	F: TGTGTCCGTCGTGGATCTGA<br>R: TTGCTGTTGAAGTCGCAGGAG
Pat2	F: AGCCACCCCTCTCAATCT<br>R: TGCCTTTGACCAGATGAACC
Pgc1α	F: AAGAGCGCCGTGTGATTTAC<br>R: TCCATTCTCAAGAGCAGCGA
Pparα	F: CCTGAACATCGAGTGTCGAA<br>R: ACGGCAGTACTGGCATTTGT
Pparγ	F: GGAAGACCACTCGCATTCCTT<br>R: TCGCACTTTGGTATTCTTGGAG
Prdm16	F: CAGCACGGTGAAGCCATTC<br>R: GCGTGCATCCGCTTGTG
Sirt1	F: GCTGACGACTTCGACGACG<br>R: TCGGTCAACAGGAGGTTGTCT
Tbx1	F: GGCAGGCAGACGAATGTTC<br>R: TTGTCATCTACGGGCACAAAG
Ucp1	F: GGCATTCAGAGGCAAATCAGCT<br>R: CAATGAACACTGCCACACCTC
